# Regulatory Effect of *let-7f* Transfection in Non-Small Cell Lung Cancer on its Candidate Target Genes

**DOI:** 10.52547/ibj.26.3.209

**Published:** 2022-04-27

**Authors:** Venus Zafari, Milad Asadi, Nasim Bakhtiyari, Mahsa Sadeghzadeh, Majid Khalili, Habib Zarredar, Soghra Bornehdeli, Ensiyeh Seyedrezazadeh

**Affiliations:** 1 Tuberculosis and Lung Diseases Research Center, Tabriz University of Medical Sciences, Tabriz, Iran;; 2 Immunology Research Center, Tabriz University of Medical Sciences, Tabriz, Iran;; 3 Department of Basic Oncology, Institute of Health Sciences, Ege University, Izmir, Turkey

**Keywords:** MicroRNAs, Biomarkers, Carcinoma, Non-Small-Cell Lung

## Abstract

**Background:**

Let-7f has essential impacts on biological processes; however, its biological and molecular functions in lung cancer pathogenesis have yet been remained unclear. We aimed to investigate the expression level of let-7f and its candidate target genes both in lung cancer tissues and A549 cell line.

**Methods:**

Bioinformatics databases were first used to select candidate target genes of let-7f. Then the relative gene and protein expressions of let-7f and its target genes, including HMGA2, ARID3B, SMARCAD1, and FZD3, were measured in lung tissues of Non-Small Cell Lung Cancer (NSCLC) patients and A549 cell line using quantitative real-time PCR and Western blotting. The electroporation method was used to transfect A549 cells with let-7f mimic and microRNA inhibitor. The impact of let-7f transfection on the viability of A549 cells was assessed using MTT assay. The expression data of studied genes were analyzed statistically

**Results:**

Results indicated significant downregulated expression level of let-7f-5p (*p* = 0.0013) and upregulated level of the HMGA2 and FZD3 in NSCLC cases (*p* < 0.05). In A549 cells, after transfection with let-7f mimic, the expression of both mRNA and protein levels of HMGA2, ARID3B, SMARCAD1, and FZD3 decreased. Also, the overexpression of let-7f significantly inhibited the A549 cell proliferation and viability (*p* = 0.017).

**Conclusion:**

Our findings exhibited the high value of let-7f and HMGA2 as biomarkers for NSCLC. The let-7f, as a major tumor suppressor regulatory factor via direct targeting genes (e.g. HMGA2), inhibits lung cancer cell viability and proliferation and could serve as a marker for the early diagnostic of NSCLC.

## INTRODUCTION

Despite scientific advances in elucidating the critical roles of genetics and the immune system in lung cancer, it has remained the most prevalent type of cancer and the leading cause of cancer-related death globally^[1]^. As a matter of fact, due to the lack of clinical symptoms and effective screening programs in primary stages, most cases are diagnosed at advanced stages^[2]^. Therefore, the development of new biomarkers for the early detection of cancer is necessary to provide an opportunity for improving the survival rate of patients^[3]^.

MicroRNAs are single-stranded non-coding and regulatory RNAs that adjust the expression of their target genes at the post-translational level. These RNAs are new candidates for serving as diagnostic biomarkers or novel therapeutic targets^[4,5]^. A singular microRNA can modulate various genes involved in diverse physiological processes and cellular events. Indeed, the oncogene or tumor suppressor role of each microRNA depends on the essence of its under influence gene^[6]^. Also, miRNAs are capable to interact with not only coding mRNAs but also lncRNAs^[7]^. Therefore, owing to the regulatory function in different ways, microRNAs have been in the spotlight to discover their exact role in varying types of cancer^[8]^. 

Let-7 is one of the major regulator families of the differentiation, pluripotency, and apoptosis in eukaryotic cells. It is the largest family comprising of 10 mature subtypes, including let-7a, b, c, d, e, f, g, I, miR-98, and miR-202, which are conserved and have a common seed sequence^[9,10]^. As revealed in studies, genes targeted by these subtypes often act as an oncogene^[11-13]^. Let-7f gene is located in 9q22.3 and is involved in a broad spectrum of physiological processes, such as angiogenesis^[14]^, immunocyte differentiation^[15]^, replicative senescence^[16]^, growth arrest^[17]^, pulmonary arterial hypertension, and carcinogenesis^[18]^. Let-7f downregulation has been reported in varied human malignancies, including nasopharyngeal carcinoma, ovarian disease, and human gastric malignant growth^[9,19]^. However, it is upregulated in primary breast cancer and hepatocellular carcinoma^[20]^. Based on evidence, the expression level of let-7f was stable or overexpressed on typical and atypical carcinoid lung cancer^[21]^. Nevertheless, the biological function and the molecular mechanisms of Let-7f in lung cancer pathogenesis remain unclear.

According to our bioinformatics outcome, the target genes for let-7f were selected on the basis of computational algorithms, including HMGA2, ARID3B, SMARCAD1, and FZD3 for let-7f. Each of these factors acts on important biological pathways whose defects lead to the destruction of the cell cycle and subsequent cell division and unrestrained. Therefore, they may be used as potential diagnostic or prognostic biomarkers or as therapeutic targets. Hence, in this study, we aimed to investigate the expression level differences of let-7f and its four candidate target genes both in human lung cancerous tissues and A549 cell line. We also checked the expression alterations of target genes after inducing let-7f in the lung cancer adenocarcinoma cell line.

## MATERIALS AND METHODS

**Patients and samples**

A total of 33 participants, composed of 17 NSCLC patients and 16 control ones, at Imam Reza and Shahid Madani Hospitals of Tabriz University of Medical Sciences (Tabriz, Iran) were included in this case-control study between April 2017 and May 2018. The subjects with hemoptysis, prior radiotherapy or chemotherapy, and tuberculosis, or patients who refuse to participate in this study were excluded. The lung tissues of participants were first collected by the bronchoscopy and needle biopsy techniques as the routine parts of the patient diagnostic approach. Then the samples were immediately placed into an RNase later solution (Qiagen, Hilden, Germany) and transferred to the laboratory. 

**Cell culture and transfection**

Human alveolar basal epithelial cell adenocarcinoma (A549) cells were purchased from the Pasteur Institute of Iran (Tehran). The cells were grown in RPMI-1640 medium supplemented with 10% fetal bovine serum medium (Gibco, Carlsbad, CA) in 95% humidity and 5% CO_2_ condition at 37 °C for 48-72 hours. All the experiments were performed when the cells reached the logarithmic phase. A number of 2 × 10^6^ A549 cells were used for transfection by the electroporation method. Transfection of 1 µg of let-7f-5p mimic (Qiagen, Hilden, Germany) and 1 µg of let-7f-5p inhibitor (as a negative control) (Qiagen, Hilden, Germany) in a 0.2-cm cuvette was performed by the Gene Pulser Xcell device (BioRad, Belgium) using 21 mM of HEPES, 37 mM of NaCl, 5 mM of KCl, 0.7 mM of Na_2_HPO_4_.7H_2_O, and 6 mM of dextrose, a single 160 V (exponential) pulse with a duration of 20 ms. 

**Total RNA isolation and reverse transcription**

The total RNAs from tissue samples and cultured cells were extracted before and after transfection using RiboEx reagent (GeneAll Biotechnology, Seoul, Korea) according to the manufacturer’s instructions. The quality and quantity of the extracted RNA were assessed by gel electrophoresis and NanoDrop OneC Spectrophotometer (Thermo Scientific™, USA). For cDNA synthesis, the reverse transcriptions were carried out using two distinctive types of kits, including the miScript II RT Kit (Qiagen, Hilden, Germany) for miRNA qPCR and 2× RT-PCR Pre-Mix (Taq) Universal cDNA synthesis kit (BioFACT ^TM^, Seoul, South Korea) for qPCR of target genes. The total RNA isolation and cDNA synthesis were conducted before and after transfection with let-7f mimic and microRNA inhibitor.

**Bioinformatics analysis**

In order to reveal potential targets of let-7f, we performed *in silico* analysis in two independent databases: miRDB (http://mirdb.org/miRDB/) and TargetScanHuman (http://www.targetscan.org). Gene MANIA (https://genemania.org) and KEGG (https://www.kegg.jp) databases were used for analyzing the relationships between genes.

**Quantitative real-time PCR**

qRT-PCR was utilized for assessing the expression level of *let-7f* microRNA and its target genes, including *FZD3, SMARCAD1, HMGA2*, and *ARID3B*. The *GAPDH* and *U6* were used as internal controls both in tissue specimens and A549 cells before and after transfection. The expression alteration of these genes was investigated after the transfection of let-7f mimic. The qRT-PCR was performed using RealQ plus 2× Master Mix Green (Ampliqon, Denmark) and Step One^TM^ qRT- PCR System (Applied Biosystem, Foster City California, USA). The primer sequences of target genes are displayed in Table 1 with more details. The primer sets of let-7f gene were purchased from Exiqon/Qiagen (product number: 202105; Hilden, Germany). In addition, the exact sequence of 3’UTR that has complementary to the miRNA seed region is displayed in Table 2.

**Western blot analysis** 

Western blot analysis was performed to discover changes in *HMGA2*, *ARID3B*, *SMARCAD1*, and *FZD3* protein levels. For this point, the samples were first lysed using RIPA buffer (Thermo Fisher Scientific™, Catalog number: 89900). In the next step, lysates were centrifuged at 13,300 ×g., Total protein concentration of the supernatants was measured using Bradford assay. Following separation by SDS-PAGE, the proteins were transferred to PVDF membranes. To block the membranes, 2% low-fat milk in Tris-buffered saline was used and incubated for 16-18 h with commercially existing *β-Actin* (C4: sc-47778), *HMGA2* (Abcam, ab207301), *ARID3B* (Abcam, ab227870), *SMARCAD1* (LSBio, LS-C80097), and *FZD3* (Abcam, ab217032) primary antibodies. In the next step, the membranes were incubated with Goat Anti-Rabbit IgG H&L (HRP) (ab6721), and bands were visualized by improved chemiluminescence. Densitometry was accomplished by the means of NIH Image/Image J software. 

**Cell proliferation (MTT) assay**

The proliferation of A549 cells was evaluated after transfection by MTT assay. A number of 10 × 10^3^ stable let-7f transfected and non-transfected control cells were seeded in 96-well culture plates. After 48-h incubation at 37 °C, 50 µl of MTT reagent (Sigma-Aldrich, St. Louis, MO, USA) with the concentration of 2 mg/ml in PBS was added to each well containing 100 µl of RPMI media. Then the plate was incubated in a CO_2 _incubator (Memmert, Schwabach, Germany) in darkness at 37 °C for 4 h. Afterward, supernatant media containing MTT solution were discarded, and the formazan crystals were dissolved by 100 µl of DMSO and shaken in 1000 RPM. The optical density of wells was measured using an ELISA reader (BioTek, ELx800, USA) at a wavelength of 570 nm. 

**Table 1:** Primer sequences used for qRT-PCR

**Target genes**	**Primer sequences**	**Annealing** ** temperature**
ARID3B-F	5′-TCCCACCACATGGACAACAAGCTA-3′	59
ARID3B-R	5′-TCATCCAGATTCCAGTTCGGCTGT-3′	
		
BZW1-F	5′-GTGTTTGCAGCCCAAGAAGAT-3′	57
BZW1-R	5′-GCTAGCTTGTTCCTCTCCGAC-3′	
		
FZD3-F	5′-GGCTGTGTCAGCGGGCT-3′	59
FZD3-R	5′-TCTTCAGGCCAAGGAACACC-3′	
		
HMGA2-F	5′-TGGGAGGAGCGAAATCTAAA-3′	59
HMGA2-R	5′‐TCCCTGGAGAAGAGCTACG‐3′	
		
SMARCAD1-F	5′‐CTACCATGGCACGTAGAAATG‐3′	59
SMARCAD1-R	5′‐CACTTCTTTGTGGAAAAAGTTCC‐3′	
		
GAPDH-F	5′-CATGGCCTCCAAGGAGTAAG-3′	58
GAPDH-R	5′-GCTTGAGCACAGGGTACTTTA-3′	
		
miRNA		**Product number**
Let-7f	Purchased from Exiqon	202105
U6	Purchased from Exiqon	218300

**Table 2:** The complementary region of let-7f sequence and 3’UTR region of target genes

**Position**	**Sequences**	**Region**
21-28 of HMGA2 3' UTR hsa-let-7f-5p	5' ...GCCAACGUUCGAUUUCUACCUCA 3' UUGAUAUGUUAGAUGAUGGAGU	8mer
		
68-74 of ARID3B 3' UTR hsa-let-7f-5p	5' ...UUGAUGCACAGAAUUUACCUCAU 3' UUGAUAUGUUAGAUGAUGGAGU	7mer
		
1873-1880 of FZD3 3' UTR hsa-let-7f-5p	5' ...AAAUACGUACUCUACCUACCUCA 3' UUGAUAUGUUAGAUGAUGGAGU	8mer
		
330-336 of SMARCAD1 3' UTR hsa-let-7f-5p	5' ...UUUUUGUAAUUAUUUCUACCUCC 3' UUGAUAUGUUAGAUGAUGGAGU	7mer

**Statistical analysis**

The data were analyzed with GraphPad Prism statistical software (Version 6.0, San Diego, CA). All results were expressed as mean ± SD with a statistical significance level of 5%. The normality distribution of variables was tested by the Kolmogorov-Smirnov test. Student t-test was used for comparing the data between case and controls and the Pearson tests for assessing the correlation between groups.

## RESULTS

**Bioinformatics analysis**

**Fig. 1:**
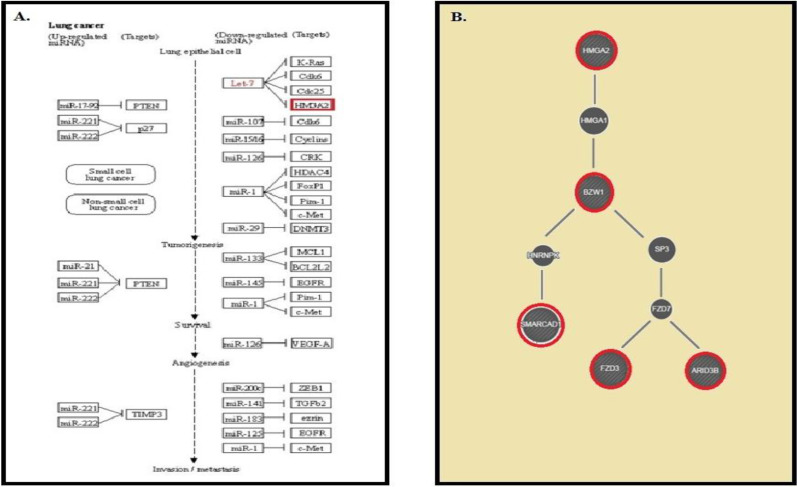
The results of (A) the KEGG database and (B) co-expression analysis by the GeneMANIA database.

According to the data obtained from databases, the *let-7f-5p* may target *HMGA2*,* SMARCAD1*,* ARID-3B*, and *FZD3* genes with prediction scores of 100, 99, 100, and 96, respectively. Based on the KEGG database analysis, *let-7f* most likely targets the *HMGA2* in NSCLC (Fig. 1A). In addition, the information of the GeneMANIA database verified the relationships between the expression of HMGA2 and four aforementioned candidate genes, which are shown in Figure 1B.

**Table 3:** General characteristic of study patients

**Characteristic**	**Case (n = 17)**	**Control (n = 16)**
Age (mean ± SD)	56.61 (16.88)	53.30 (16.48)
		
Sex, n (%)		
Male Female	12 (71) 5 (29)	9 (56) 7 (44)
		
Type of cancer, n (%)		
Adenocarcinoma Squamous Large Cell	6 (35) 7 (41) 4 (23)	- - -
		
Stage of cancer, n (%)		
I II III IV	1 (5.9) 2 (11.8) 11 (64.7) 3 (17.6)	- - - -

**Patient’s characteristics**

The general characteristics of the participants are shown in Table 3. From 33 subjects, 17 were diagnosed as NSCLC cases, including eight adenocarcinomas, 10 squamous cells, and seven large cell cancerous cases. Also, 16 of them were detected as noncancerous controls. The frequency of each stage of NSCLC patients are reported in Table 3.

**Downregulation of let-7f expression and overexpression of its candidate target genes**

**Fig. 2:**
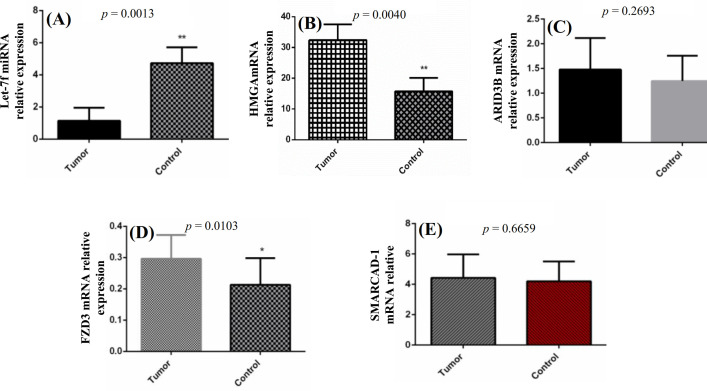
Relative expression of let-7f and its potential target genes in tumor and control tissues of NSCLC patients.

Statistical analysis of mRNA expression level of *let-7f* and the predicted target genes in lung tissue samples exhibited the downregulated levels of *let-7f* in cases compared to the controls (*p* = 0.0013). However, the expression level of target genes, including *HMGA2* and *FZD3*, in tumor tissues was overexpressed in comparison to the normal tissues (*p* < 0.01). A nonsignificant difference was found in the expression level of *ARID3B* and *SMARCAD1* between the tumor and control tissues (*p *> 0.05; Fig. 2).

**Downregulated mRNA levels of target genes**

**Fig. 3:**
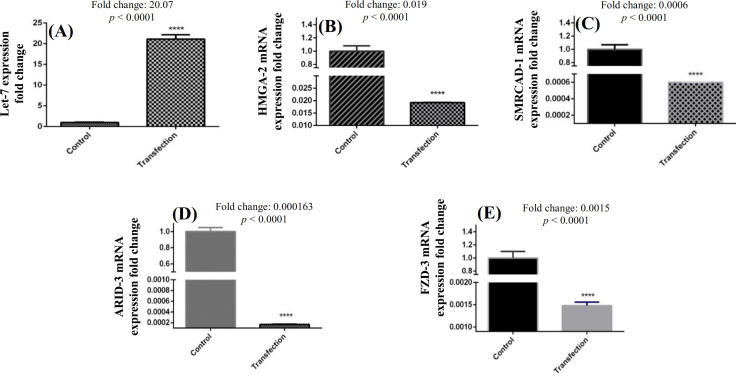
Alteration of gene expression levels of *let-7f* (A) and potential target genes (B, *HMGA2*; C, *SMARCAD1*; D, *ARID3*; E, *FZD3*) after transfection with let-7f mimic.

qRT-PCR analysis of stable *let-7f* expressing A549 cell lines, in which the level of let-7f elevated as a result of transfection, indicating the downregulated expression levels of *HMGA2*, *ARID3B*,* SMARCAD1*, and* FZD3* in comparison to untransfected and negative control cells (*p* < 0.0001). Figure 3 shows the results of this section with more details.

**Downregulated protein levels of target genes**

**Fig. 4:**
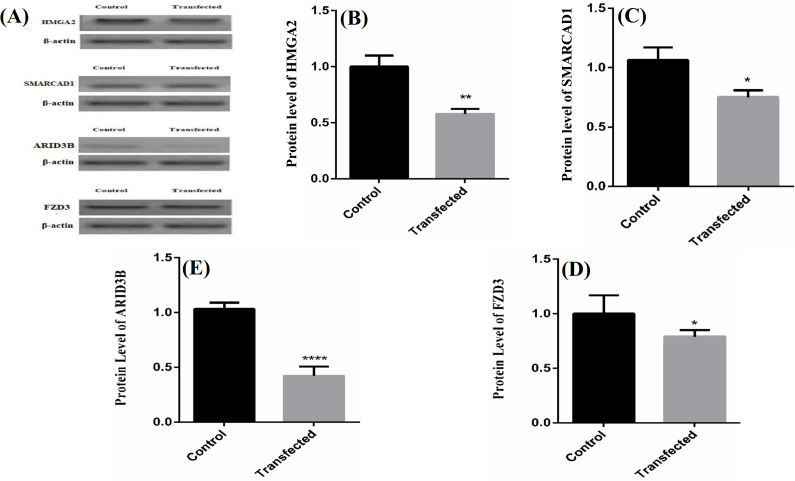
The alteration of potential target protein expression levels of (A) western blotting, (B) HMGA2, (C) SMARCAD1,  (D) ARID3, and (E) FZD3) after transfection with let-7f mimic.

Analysis of protein expression by Western blotting in transfected A549 cell lines indicated a significant decrease in *HMGA2*,* ARID3B*,* SMARCAD1*, and* FZD3* expression levels as compared to untransfected cells (Fig. 4).

**Reduced viability of the lung cancer cells by Let-7f**

**Fig. 5:**
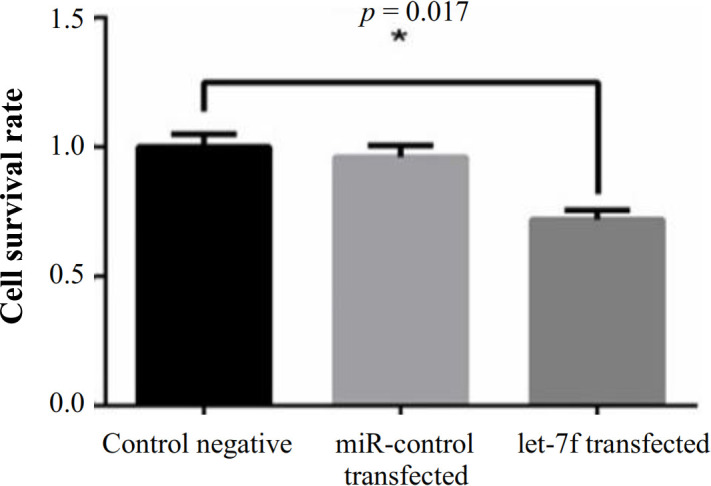
The cell survival rate of A549 cells after transfection with let-7f mimic and miR-control.

To investigate whether *let-7f* transfection affects the viability of the lung cancer cells (A549), we performed the MTT assay, which showed that an increased level of *let-7f* expression negatively affects the cell viability in A549 cells (*p* = 0.017). Figure 5 represents the alterations of viability after transfection with *let-7f* mimic and microRNA inhibitor.

**Correlations of the expression level of studied genes**

**Fig. 6:**
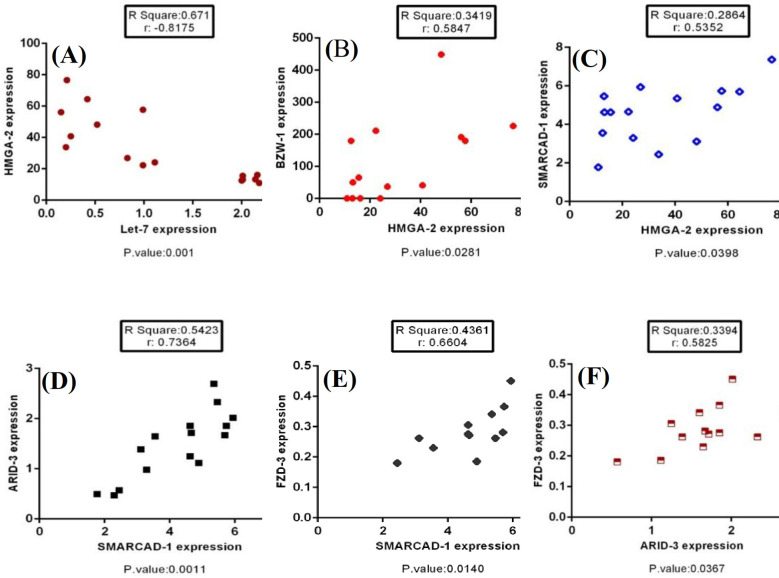
Significant relationships between the expression level of let-7f and its target genes (*p* < 0.05). (A), let-7f and HMGA1; (B) BZW1 and HMGA2; (C), SMARCAD1 and HMGA2; (D), ARID3B and SMARCAD1; (E), FZD3 and SMARCAD1; (F), FZD3 and ARID3B.

According to the analysis of the data, significant correlations were found between the expression levels of *let-7f* and *HMGA2* (r = -0.81, *p* < 0.05). Figure 6 depicts such correlations in detail. 

**Investigation of the potential of genes as a diagnostic biomarker**

**Fig. 7:**
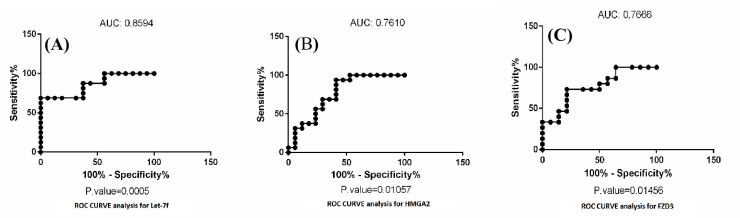
The ROC curve automatically generated from 36 points of cut-off values. The AUCs were 0.85, 0.76, and 0.76 for let-7f, HMGA2, and FZD3 genes, respectively.

ROC curves were plotted for both let-7f and its target genes. Later, the AUC was computed to assess the specificity and sensitivity of markers to anticipate NSCLC incidence. In other words, the plot shows the specificity and sensitivity of biomarkers at different cut-off points. The ROC area for *let-7f*, *HMGA2*, and *FZD3* were 0.86, 0.76, and 0.76, respectively, and the differences were significant (*p* < 0.01; Fig. 7). The other target genes had not any significant AUC.

## DISCUSSION

MiRNAs, which their roles in the genesis and development of cancer have been proven in several studies, possess a particular ability to modulate the complete network of gene regulation in a number of cancer-related pathways^[9]^. 

Considering the let7f implication in many cancers initiation and progression, we chose let-7f and its candidate targets to investigate the molecular mechanism of its involvement in NSCLC. According to the outcome of this study, the significant downregulated level of let-7f was shown in the lung tissue of NSCLC patients compared to the control participants, suggesting the tumor suppressor effect of let-7f-5p in lung cancer. The results of our project are consistent with previous studies that had shown the downregulation of this gene in various malignancies, including gastric cancer^[10]^, papillary thyroid^[11]^, glioma^[12]^, ovarian^[13]^, and head and neck cancer^[14]^. However, some types of cancer, e.g. primary breast cancer and hepatocellular carcinoma, indicated the upregulation of let-7f in tumor cells compared to normal non-cancerous cells^[15,16]^. According to our study, induction of the expression of let-7f-5p in A549 cells, through its mimic transfection, led to the elevation of the let-7f expression and followed by the downregulation of its target gene expression, including HMGA2, SMARCAD1, FZD3, and ARID3B. These alterations demonstrated that the mentioned genes may be directly regulated by let-7f, which is probably due to the presence of complementary sequences. HMGA2, which had a significant negative correlation with the let-7f expression, is a factor that controls the cell cycle. According to evidence, HMGA2 promotes and accelerates cancer cell proliferation through Cyclin A, Cyclin E, Cyclin D1, Cyclin B2^[17]^. Furthermore, after let-7f transfection, the viability of the lung cancer cells decreased, which can confirm the inhibitory role of this gene in cancer pathogenesis. In the present study, the possibility of using let-7f and its target genes as diagnostic or prognostic biomarkers was evaluated by analyzing ROC curves. Our data suggest let-7f as a powerful biomarker, followed by HMGA2 and FZD3. Similarly, Shee *et al.*^[18]^, who have investigated the capability of the let-7f as a biomarker for non-muscle invasive bladder cancer, have stated that let-7f can be used as a prognostic biomarker, and the expression level of this gene in bladder biopsies and also plasma has a positive and significant relationship with the risk of recurrence^[18]^.

Most scientists are trying to discover the molecular mechanism by which let-7f plays its inhibitory role during the process of carcinogenesis. Research by Liang et al.^[10]^ on gastric cancer cells has shown that let-7f reduces the invasion and metastasis of this type of cancer by targeting genes involved in metastasis, such as MYH9. According to the result of Yan *et al.*’s^[12]^ study on glioma cell lines, let-7f via targeting MMP2 and MMP9 restricts the invasion and migration ability of glioma tumor cells. Moreover, let-7f targets cell cycle checkpoint factors such as Cyclin D1, Cyclin E, P27, and P21, thereby induces the cell cycle arrest and inhibits cell proliferation and colony formation. In renal cell carcinoma, the luciferase assay method demonstrated that let-7f targets the KLK10, a subgroup of the Kallikreins family that is expressed in various normal organs such as breast, testis, ovary, kidney, and prostate with a diverse set of physiological functions^[19,20]^. As the precise molecular mechanisms of regulatory roles of let-7f in NSCLC have yet remained unclear, in this study, we analyzed not only the let-7f deregulation in lung cancer but also the impact of its overexpression on the candidate genes, hoping to understand its possible mechanism of action. Nevertheless, to achieve the desired goal, some additional studies are needed to determine the exact molecular activity mechanism of let-7f in NSCLC. Determination of the functional mechanism of pivotal molecules like let-7f will lead to the discovery of new therapeutic strategies targeting defective molecules. It may be noted that NSCLC subgroup of lung cancer has not been studied in previous studies. Besides, earlier investigations have examined the whole let-7 family^[22,23]^, while the present study has focused on the let-7f member of the let-f family and also has assessed both HMGA2 and three of the strongest candidate target genes of let-7f. Our study had some limitations such as the small number of NSCLC tissues as a result of sampling difficulty (lung biopsy)

In summary, the let-7f, as a major tumor suppressor regulatory factor, can exert its role in lung cancer via direct targeting genes such as HMGA2, SMARCAD-1, ARID-3, and FZD3 and can affect important processes, e.g. viability and proliferation of cancer cells. This gene can also benominated as a biomarker for NSCLC.

## DECLARATIONS

### Ethical statement

The above-mentioned sampling protocols were approved by the Ethical Committee of Tabriz University of Medical Sciences, Tabriz, Iran (ethical code: IR.TBZMED.REC.1397.060). Written informed consents were obtained from all participants.

### Data availability

The analyzed data sets generated during the study are available from the corresponding author on reasonable request.

### Author contributions

All authors contributed to the study conception and design; ES and VZ were involved in the methodology of study and performed data analysis and interpretation; AS participated in patient selection; VZ, MS, and SB involved in cell cultures, proliferation, and transfection; VZ, MA, HZ, and NB were involved in gene and protein analysis; MA and VZ participated in bioanformatic analysis; ES, VZ, and KK assisted in manuscript edition, developed the drafted and finalized the manuscript; each listed author approved the final version submitted for publication.

### Conflict of interest

None declared.

### Funding/support

This study was supported by the Non-Communicable Center of the Ministry of Health and Medical Education, and Tuberculosis and Lung Disease Center of Tabriz University of Medical Sciences (Tabriz, Iran. of Medical Sciences.
